# The Impact of China’s Zero Markup Drug Policy on Hospitalization Expenses for Inpatients in Tertiary Public Hospitals: Evidence Based on Quantile Difference-in-Difference Models

**DOI:** 10.3390/healthcare9070908

**Published:** 2021-07-18

**Authors:** Ziling Ni, Jie Jia, Lu Cui, Siyu Zhou, Xiaohe Wang

**Affiliations:** Department of Health Service Management, School of Public Health, Hangzhou Normal University Hangzhou Normal University, Hangzhou 311100, China; ziling@hznu.edu.cn (Z.N.); jj942431027@163.com (J.J.); blp1887@163.com (L.C.); siyuzhouhz@126.com (S.Z.)

**Keywords:** Zero Markup Drug Policy, hospitalization expenses, QDID, tertiary hospitals

## Abstract

Objectives: The aim of this study was to determine the impact of the Zero Markup drug (ZMD) policy on hospitalization expenses for inpatients in tertiary Chinese hospitals. Methods: Using the administrative data from hospital electronic health records (EHRs) between 2015 and 2017, we implemented the quantile difference-in-differences (QDID) estimators to evaluate the impact of the ZMD policy on hospitalization expenses while controlling for patient-level and hospital-level characteristics. Results: According to the QDID models, the introduction of ZMD policy significantly induced lower drug costs for all inpatients especially at the 50th (-USD 507.84 (SE = USD 90.91), 75th (-USD 844.77 (SE = USD 149.70), and 90th (-USD 1400.00 (SE = USD 209.97)) percentiles of the overall distributions. However, the total hospitalization, diagnostic, treatment, material and services expenses for inpatients were significantly higher for the treated group than the control group. This tendency was more pronounced for inpatients in tertiary hospitals with lower expenses (in the 10th, 25th and 50th percentiles). Conclusion: The implementation of ZMD policy alone may not be enough to change the medical service providers’ profit-driven behavior. The targeted supervision of hospital costs by the Chinese health administration department should be strengthened to avoid unreasonable hospital charges.

## 1. Introduction

In the past decades, public hospitals in China were allowed by the government to charge 15% or more for prescribed medicines based on their procurement prices, named as the Fixed Percent Markup (FPM), to balance the financial loss due to the decline of government’s subsidies for public hospitals and to compensate facilities for losses from providing services priced below cost to ensure the sustainable development of public hospitals [1].The policy was pushed toward public hospitals to increase revenues through shifting priorities from consultation services to prescriptions [2]. However, this policy gradually evolved into a profit-seeking mechanism, leading to inappropriate behavior, such as over-prescription, over-infusion, and abuse of antibiotics. Physicians tend to prescribe expensive drugs or to overprescribe unnecessary drugs to patients in order to gain profits [3]. For a long time, drug expenses have been growing by 15% per year, and drug revenue has become the foremost income source, covering 40% or more of public hospitals’ budget [4,5]. By 2007, sales of medicines still represented more than 41% of hospital revenues, and drug expense accounted for 42.7% of total health care expense per inpatient episode [6,7].

To lower pharmaceutical expenses and maintain the affordability of medical care, a series of policies for the pharmaceutical reform was launched by the Chinese government. To decrease the inflated drug price and eradicate the perverse incentives for the overuse of drugs, the zero markup for drugs (ZMD) policy was initially introduced in China in 2009, along with the National Essential Medicine Policy, which required that primary care institutions including township and villages health institutions only can prescribe essential medicines and sale drugs with no markup for patients [8]. To reduce the medical expense burden of more patients and eradicate the perverse physicians’ incentives for the overuse of medicines, the ZMD policy was later gradually extended to county hospitals in phases between 2012 and 2015. Additionally, the Chinese government required that all the public tertiary hospitals in China should be covered by the zero-markup policy on the sale of medicines by the end of 2017.

Under the policy, public tertiary hospitals can only sell all the drugs (except for traditional Chinese drugs) at purchase price without any markup. As drug revenues will be substantially reduced, the government increased the government subsidies and allowed hospitals to compensate for the loss of profits by raising the price of medical services [9]. As distinct from primary health care facilities, who obtained full budgetary support from the government, public tertiary hospitals were only able to obtain a small amount of government financial subsidy (accounting for 10% of the loss of profits caused by the policy) and had to compensate for the loss of profits by depending on adjusted (increased) fee levels on other items [1]. With the removal of the profit margin for drug sales, the incentives of physicians to overprescribe was reduced. However, the loss of drug revenues caused by the reforms also left big holes in some hospital budgets [10]. Meanwhile, the reform resulted in potentially inappropriate provider revenue structures, such as irrational prescription service fees, potentially boosting patients’ medical costs [11].

After implementation of the ZMD policy as an important measure for deepening the reform of the medical and health system in China, experiences and problems arising from local implementation need to be collected. Extensive studies conducted in the primary healthcare facilities or county hospitals have established that the ZMD policy change led to a reduction in drug expenditures, and an increase or no measurable changes in total health expenditures [12,13,14]. Data from these studies suggest that primary healthcare facilities or secondary public hospitals, who had a greater reliance on drug revenue before the reform, were able to offset reductions in drug revenue through increases in other sources [10,11,12,13,14,15]. After a comprehensive analysis of the existing research, it was found that although the previous studies provided some evidence of the consequences of China’s ZMD policy in primary healthcare facilities and secondary hospitals, the empirical evidence remains limited on the effect of ZMD policy in the tertiary public hospitals on inpatients’ expenses, because most of the previous studies were conducted on the basis of simple descriptive analyses, which lack rigorous design in evaluating the policy. In addition, almost all of the studies analyzed the changes in the medical expense in the institution, and few of the studies on the impact of ZMD policies on patients’ expenses controlled for variables like age, gender, admission status, insurance status, and severity of medical condition.

Furthermore, it has now been well established by a variety of studies that the effect of a certain policy on healthcare expenses and use can be different in the lower medical expenditure subpopulations compared to higher medical expenditure subpopulations [16,17]. It has therefore been postulated that traditional regression methods (e.g., ordinary least square), which produce a single rate (i.e., the average) of change as indicated by the regression coefficient of each explanatory variable may be incapable of accurately describing the relationships between the explanatory variable and the outcome (i.e., cost) across the entire cost distribution in the population [18,19].

Consequently, the object of this study is to examine the impact of the ZMD policy on hospitalization expenses for inpatients by employing the difference-in-difference (DID) methods in conjunction with quantile regression models in the tertiary public hospitals and to examine the trends in hospitalization expenses over time while taking patient-level characteristics into consideration. 

## 2. Materials and Methods 

### 2.1. Data Collection 

This study was conducted in Shanxi province, northern China, which was ranked 21st for GDP per capita from a sample of 31 provinces in 2020. As a major energy province, Shanxi province plays an important role in the country’s economic and social development. There were 11 areas in its jurisdiction and 36.82 million residents in Shanxi, with 56.21% living in urban areas in 2016. The total health expenditure in Shanxi accounted for 7.48% of its gross domestic product (GDP) [20]. According to 2016 statistics from the National Health and Family Planning Commission, there were 49 tertiary hospitals in the province. The 49 tertiary hospitals commenced the ZMD reform in sequence over 2 stages: 22 (in cities of Taiyuan, Yangquan, Jinzhong, etc.) started in November 2016, and the remaining 27 hospitals (15 provincial hospitals and 12 municipal hospitals in the cities of Datong, Linfen, Jincheng, etc.) implemented the reform in July 2017. To ensure the consistency of the included hospitals, all municipal hospitals were included in this study while provincial hospitals were excluded.

We employed a quasi-natural experiment design with a quantile difference-in-differences (QDID) approach in this study. The QDID results reflect the differences between the two groups of hospitals between November 2015 and June 2017.The pre-intervention period was from 1 November 2015 to 31 October 2016, while the post-intervention period was from 1 November 2016 to 30 June 2017. The pre–post reform changes made by the 22 public tertiary municipal hospitals (test group) that implemented the reform in November 2016 were compared with those of the 12 public tertiary municipal hospitals (control group) that implemented the reform in the second stage (July 2017). We collected the information of inpatients with AMI and pneumonia, who were hospitalized between 1 November 2015 and 30 June 2017. This allowed us to study inpatients both before and after policy implementation. In addition, the primary reason we choose AMI and pneumonia is that they are common conditions in China. [21] 

Our primary concern was the impact of the ZMD policy reform on the hospitalization expenses for inpatients in the public tertiary hospitals. We used administrative data collected through hospital electronic health records (EHRs), certified by the Medical Record Management Association of the Chinese Hospital Association. EHRs in these hospitals follow a national template and have adopted standardized disease coding according to the International Classification of Diseases, Tenth Revision (ICD-10). The study members integrated EHRs from all 34 tertiary municipal hospitals into a single database, which included information about both the patient-level and hospital-level characteristics. All personal identifiers of patients and medical personnel (e.g., name, ID card number, and/or insurance number) were excluded prior to the commencement of the study. Patient-level characteristics included the following variables: patient socio-demographic characteristics (e.g., age, gender, race/ethnicity); diagnosis codes (i.e., primary diagnosis code and up to 10 secondary diagnosis codes); up to seven procedure codes; total cost; service charges in subcategories; length of stay (LOS); outcomes (such as discharge status and adverse medical events during hospitalization); and so on. 

To ensure accurate estimation of processes and outcomes, inpatients were excluded if they had missing information for any of the case mix adjustment variables, or if they were transferred out. Inpatients whose hospitalization expenses were less than ¥100 (USD 14.49) were excluded as well. In addition, inpatients discharged from the hospital within one day after admission were excluded because of the limited treatment time. Furthermore, for a more stable analysis, hospitals with fewer than 10 pneumonia or AMI inpatients within the proposed timeframe were excluded [21,22].

### 2.2. Measures

To estimate the relationship between the ZMD policy and hospitalization expenses, the total hospitalization expenses per inpatient incurred during hospitalization were calculated as the aggregate of all fees for healthcare service, including drugs, treatments, sanitary materials, and diagnostics. We included patient-level and hospital-level variables as controls. Patient-level variables extracted from the database included gender, age, major diagnostics, admission source, admission status, comorbid conditions (summarized using severity-weighted Charlson Comorbidity Index (CCI)), length of stay (LOS), and type of insurance coverage. Major diagnoses were based on the following ICD-10 codes: J10.x-J18.x for patients with pneumonia and I21.0, I21.1, I21.2, I21.3, I21.4, I21.9 for patients with AMI. The admission source was defined in the EHRs as outpatient medical service, emergency medical service, referrals, and other sources. Admission status, used as a proxy for disease severity, was categorized into three types: regular, urgent, and critical. CCI was calculated using the ICD-10 codes of comorbidities based on the studies of Quan and colleagues to reflect comorbidity effects [22,23]. LOS was defined as the number of inpatient days, from the day of admission until discharge. In this study, population, patients’ health insurance included the urban employee-based basic medical insurance (UEBMI), urban resident-based basic medical insurance (URBMI), the new cooperative medical scheme (NCMS), self-payment, and other.

### 2.3. Statistical Analysis

The descriptive statistics of patient-level and hospital-level variables included mean, standard deviation (SD), and quartiles for the continuous variables, and numbers and percentages for the categorical variables. Analysis of variance (ANOVA) and a Kruskal-Wallis test were used to test the significance of difference among the continuous variables. Pearson’s χ^2^ and Fisher’s tests were used to compare the categorical data.

Since this study focuses on identifying the distributional impact of the ZMD policy and other determinants of hospitalization expenses, we investigated the impact of implementing the ZMD policy on hospitalization expenses for inpatients using the DID and QDID method. 

We conducted DID models using generalized linear regressions since the outcome indicators did not follow a normal distribution.

$$y_{ikt}=\alpha_{0}+\beta \times ZMDP_{ikt}+\gamma \times X_{kt}+\alpha_{k}+\delta_{t}+\varepsilon_{ikt}$$

where *i* denotes the specific hospitalization expense, *k* indicates the tertiary hospital, and *t* indicates the month. $\alpha_{k}$ is a series of fixed effects of individual hospitals that control for the unobserved time-invariant individual heterogeneity across hospitals. δ_t_ represents month dummies used for controlling for the flexible time effects. $\varepsilon_{ikt}$ refers to the error term.

This detailed quantile regression was used to estimate the conditional percentiles of total cost and association within the time period while controlling for patient and hospital factors. We performed DID analyses using quantile regressions:

$${∆}^{QDID}=F_{Y_{01}}^{-1}\left(q\right)-F_{Y_{00}}^{-1}\left(q\right)=F_{Y_{01}}^{-1}\left(F_{Y_{01}}\left(y\right)\right)-F_{Y_{00}}^{-1}\left(F_{Y_{00}}\left(y\right)\right)$$

The implementation of the QDID estimator requires running the DID estimator in each quantile *q*, with the coefficients α_q_, β_q_ and γ_q_ indexed by the specific quantile. Specifically, for any fixed γ, the quantile q for y in the distribution of Y_10_ (outcome for the treated group in first (pre-ZMD) period) is obtained as q = F_Y10_ (y). To obtain the counterfactual value for $Y_{11}^{0}$ at the quantile q, the difference in outcome for the control group post- and pre-ZMD implementation [24]. Quantile regression for total hospitalization expenses was performed from the 10th to 90th percentiles (i.e., Q10 = 0.10, Q25 = 0.25, Q50 = 0.50, Q75 = 0.75, Q90 = 0.90). 

All statistical analyses were conducted using Stata version 14.00.

## 3. Results

### 3.1. Descriptive Statistics—Inpatient Characteristics

The study sample size was 16,200 inpatients, of whom 6163 were admitted at seven hospitals that had implemented the policy in the first stage and 10,037 inpatients admitted at eight hospitals where the policy had been implemented in the second stage. Table 1 provides descriptive statistics for the inpatients. All characteristics, including age, gender, disease type, admission source, admission status, CCI, type of insurance, and LOS were provided for the two groups in the two periods. 

### 3.2. Descriptive Statistics—Outcome Measures

Table 2 lists the descriptive statistics for the outcome measures. The seven measures (mean, standard deviation, 10th, 25th, 50th, 75th, and 90th percentiles) of outcomes (total hospitalization expenses, drug expenses, diagnostics expenses, treatment expenses, service expenses, and material expenses per inpatient) were reported separately by time period (whether pre- or post-intervention period) and by treatment group status (whether the inpatients belonged to hospitals that had introduced the ZMD policy in the post-intervention period). The mean total hospitalization expenses for the control group in the post-intervention period were lower than in the pre-intervention period (USD 3897.23 with a SD of USD 4901.43 vs. USD 3914.84 with an SD of USD 4173.89), while those for the treated group post-intervention were higher than pre-intervention (USD 4124.14 with a SD of USD 3787.42 vs. USD 4016.77 with a SD of USD 3454.34). The mean drug expenses for inpatients in both the control group and treated group declined post-intervention (control group: USD 1091.45 with an SD of USD 1335.68 vs. USD 1180.47 with a SD of USD 1527.3 pre-intervention; treated group: USD 1151.49 with an SD of USD 1416.49 vs. USD 1305.78 with a SD of USD 1530.12 pre-intervention). Furthermore, the reported drug cost distribution percentiles indicated that with increasing expenses, the gap for the treated group between the two periods expanded. For the control group, the diagnostics expenses were lower post-intervention than pre-intervention (USD 700 with a SD of USD 538.79 vs. USD 724.79 with a SD of USD 580.71), while those for the treated group increased post-intervention (USD 723.73 with a SD of USD 639.32 vs. USD 714.45 with an SD of USD 575.97). Post-intervention treatment expenses were higher than in the pre-intervention period for both the control group and the treated group. According to percentile distributions, the treatment expenses for control and treated groups in the two periods were similar. Mean material expenses for control groups increased post-intervention, while expenses for the treated group declined post-intervention.

### 3.3. Results Based on QDID and DID Models

Table 3 presents the estimates of the ZMD policy effect on hospitalization expenses for inpatients in the tertiary hospitals, using QDID models. The QDID estimates were adjusted for gender, age, admission status, admission source, CCI, and type of insurance coverage, which are reported as factors influencing hospitalization expenses for inpatients in previous studies. Estimates for all the inpatients shown in Table 3 indicate that compared to the control group, the treated group had higher overall hospitalization expenses (USD 2308.83 (SE = USD 476.41)), lower drug expenses (USD 661.84 (SE = USD 202.80)), higher treatment, diagnostics, and service expenses (USD 206.74 (SE = USD 78.78), USD 515.74 (SE = USD 99.24), USD 75.06 (SE = USD 24.98), respectively) following the introduction of the ZMD policy. These results are similar to those of the QDID models in terms of the signs and statistical significance of the coefficient estimates, showing the robustness of our results.

According to estimates of the ZMD policy’s effects based on QDID models, policy introduction induced significantly lower drug expenses for all inpatients, especially at the 50th (−USD 507.84 (SE = USD 90.91), 75th (−USD 844.77(SE = USD 149.70), and 90th (−USD 1400.00 (SE = USD 209.97)) percentiles of the overall distributions. However, the total hospitalization expenses for inpatients were significantly higher for the treated group than for the control group. This tendency was more pronounced for inpatients in tertiary hospitals with lower expenses (in the 10th, 25th and 50th percentiles (USD 988.32 (SE = USD 187.31), USD 1805.68 (SE = USD 251.12), USD 1141.81 (SE = USD 324.01), respectively)) as well. For inpatients, the increase in diagnostics expenses mainly occurred among inpatients with lower expenses (in the 10th (−USD 338.30 (SE = USD 60.26) and 25th percentiles (−USD 247.09 (SE = USD 54.21)). The ZMD policy led to significant increases in treatment and material expenses for inpatients especially at the 10th, 25th, and 50th percentiles of the overall distributions in tertiary hospitals. Moreover, positive DID coefficients increased significantly with the increase in treatment and material expenses, showing the robustness of our results. 

The time series analyses were conducted to compare the secular trends of the two groups of hospitals before November 2016 when the ZMD policy were not implemented in both groups. There was no statistical difference between the two groups in secular trends (*p* = 0.169, *p* = 0.528, respectively, for trends in drug expenses and total hospitalization expenses). In addition, the results of DID models are similar to the QDID models in terms of the signs and statistical significance of the coefficient estimates, showing our primary findings were unlikely to be caused by selection bias.

## 4. Discussion 

Based on administrative data from 15 tertiary hospitals in Shanxi, China, this study sought empirical evidence using QDID models to determine the effects of the ZMD policy on hospitalization expenses for inpatients, focusing on AMI and pneumonia patients. Our findings suggest that the ZMD policy did reduce drug expenses for inpatients; however, it did not lower the total hospitalization expenses per inpatient. Important differential effects were observed across the cost distribution. 

Compared with previous studies, our study has several strengths. Firstly, given the data we used in this study were obtained from November 2015 to November 2017, we were able to accurately reflect changes in hospitalization expenses before and after the ZMD policy implementation. Secondly, in addition to the total hospitalization expenses, our study also shows that drug, diagnostic procedures, treatment, material, and service expenses were higher after the implantation of the ZMD policy. To the best of our knowledge, this information has not been previously reported. The previous studies of the impact of the ZMD policy mainly focused on primary health institutions and the scope was restricted in national essential drugs. However, the ZMD policy in Shanxi was expanded to any drug sold there and all levels of health institutions including tertiary hospitals. Thirdly, given the skewed distribution of the expenses data and the cluster effects, we used the quantile regression models instead of the conventional ordinary linear regression model. Thus, our estimates are less biased. Since the QDID models show more information, the changes in the cost of patients in different subgroups that other studies cannot demonstrate can be reported in this study. Fourthly, we used administrative data from a general population comprising inpatients with pneumonia and AMI that reflected inpatient and hospital characteristics. In our study, our sample captured the diversity of patient-level and hospital-level factors on hospitalization expenses. Additionally, we chose a natural experiment that allowed us to simultaneously address the primary limitations in the empirical literature on the ZMD policy to date: the lack of a well-defined control group [25,26].

Similar to other previous studies [27,28,29,30,31], the results of this study showed that the patient characteristics (such as age and gender), the severity of the condition of the inpatients (such as CCI scores, admission status, admission sources, and LOS), and the hospital characteristics had significant relationships with hospitalization expenses in varying degrees.

Theoretically, with the introduction of the ZMD policy, drug retail will decline as a result of the drug expenses per patient, and the proportion of drug expenses in relation to total hospitalization expenses will be reduced. In the past decade, the ZMD policy for essential drugs has achieved a certain degree of success in reducing the burden on patients. There were many studies demonstrated that ZMD policy for essential drugs reduced the medical expense significantly in primary facilities and second public hospitals [11,32,33,34,35]. However, we have different findings when studying the impact of ZMD policy on hospitalization expenses for per inpatient in tertiary public hospitals. After adjusting for the effects of patient characteristics, and complexity of patients, we found that although with the ZMD policy introduction of drug expenses for patients with pneumonia and AMI were reduced, diagnostic, treatment, material, and total hospitalization expenses were all increased. The possible explanations for these different findings was that the previous studies were mainly conducted in the primary health care facilities which provided relatively simple care service for patients with common diseases [36]. The primary health care facilities cannot make up for the loss of drug markups by raising the cost of examinations, materials and operations (treatment) as tertiary hospitals do [37]. In China, the tertiary hospitals are with bigger size, higher of medical technology, more advanced medical equipment, and higher charging standards than secondary hospitals [22]. Therefore, there are more chances for tertiary hospitals to increase other medical expenses to compensate for the loss of hospitals’ drug profits. 

It is worth noting that the increase in total hospitalization expenses due to the increase in diagnostic, treatment, and material expenses mainly occurred among patients with lower expenses (in the 10th, 25th, and 50th percentiles). One possible explanation for this finding is that, compared with patients with milder conditions, patients with more serious conditions usually have higher expenses, and they actually need more examinations and treatments. Therefore, there was limited room for growth in terms of the examination and treatment expenses for this group of people. The increase in diagnostics, treatment, and material expenses after controlling for the patient characteristics, such as the patients’ comorbidity admission status, indicated that the increase in inpatients’ demand for medical services was not caused by the patients’ conditions themselves but by the providers. The reduced profits from eliminating drug markup were passed on to other medical treatments in the secondary and tertiary hospitals, so the patients’ cost burdens have not been alleviated. According to the ZMD policy, in order to ensure protection for the interests of medical service providers, the medical service fee that reflects the doctor’s service value has to increase as drug expenses decrease. Therefore, the increase in treatment expenses is relatively reasonable. However, the results of our research showed that the increase in service expenses reflecting the value of doctors’ services was too low (the coefficient values were low) to compensate for the loss of profits caused by the elimination of drug markup. Furthermore, eliminating drug markup means that the pharmacy department now brings not only benefits to the hospital, but also high running expenses [38]. Thus, supplier-induced demand will frequently, lead to over treatment and rising medical expenses.

In general, the ZMD policy is a reform that has changed unreasonable medical fee structures to avoid unreasonable increases in medical expenses in order to reduce patient expense burdens. Our study results suggest that it is very necessary to establish a reasonable pricing system for medical service considering the affordability of finance, patients, medical insurance institutions, and hospitals. In addition, the adjustment of the price of medical services should follow the principle of improving the price of medical services that embody the value of technology and labor.

## 5. Conclusions

This article presents new evidence on the effect of China’s ZMD policy on the hospitalization expenses for inpatients. Based on the current study, we can draw several conclusions. The policy promoted the reduction of drug expenses for inpatients by controlling both the patient-level and hospital-level characteristics. However, total hospitalization expenses, including diagnostic, treatment, and material expenses for inpatients—especially inpatients with lower hospitalization expenses (in the 25th and 50th percentiles)—in tertiary hospitals increased. Therefore, the supervision of hospital expenses by the Chinese health administration department should be strengthened. Finally, policies should be implemented to avoid unreasonable charges to patients and to compensate the loss of hospitals’ drug profits. 

There are several limitations to this study. The sample came from Shanxi Province, which economic development and healthcare resource are below the national average. Shanxi province is a highly polluted region, as the mining and steel industries are highly concentrated in the region, and provide the main source of revenue for the local government. The composition of patients with respiratory disease may be different from other regions. Our findings may not be generalizable to populations living in the developed regions of China. We evaluated the ZMD policy impact in a relatively short period. Future research is needed to evaluate its long-term effects. 

## Figures and Tables

**Table 1:** Descriptive statistics of the patient characteristics during pre- and post-treated period.

	Pre-Intervention Period			Post-Intervention Period		
	Control Group	Treated Group	Total	Control Group	Treated Group	Total
Gender						
Male, *n* (%)	3472(68.77)	2138(66.13)	5610(67.74)	3523(70.63)	1909(65.15)	5432(68.60)
Female, *n* (%)	1577 (31.23)	1095(33.87)	2672(32.26)	1465(29.37)	1021(34.85)	2486(31.40)
Age						
≤40, *n* (%)	405(8.02)	209(6.46)	614(7.41)	407(8.16)	237(8.09)	644(8.13)
41–60, *n* (%)	1918(37.99)	933(28.86)	2851(34.42)	1888(37.85)	869(29.66)	2757(34.82)
>60, *n* (%)	2726(53.99)	2091(64.68)	4817(58.16)	2693(53.99)	1824(62.25)	4517(57.05)
Disease Type						
AMI, *n* (%)	1700(33.67)	1673(51.75)	3373(40.73)	1615(32.38)	1530(52.22)	3145(39.72)
Pneumonia, *n* (%)	2249(44.54)	1560(48.25)	3809(45.99)	3373(67.62)	1400(47.78)	4773(60.28)
Admission Source						
Outpatient, *n* (%)	1940(38.42)	1367(42.28)	3307(39.93)	2183(43.77)	1273(43.45)	3456(43.65)
Emergency, *n* (%)	3065(60.71)	1664(51.47)	4729(57.10)	2722(54.57)	1500(51.19)	4222(53.32)
Transfer, *n* (%)	29(0.57)	50(1.55)	79(0.95)	65(1.30)	108(3.69)	173(2.18)
Other, *n* (%)	15(0.30)	152(4.70)	167(2.02)	18(0.36)	49(1.67)	67(0.85)
Admission Status						
Regular, *n* (%)	3115 (61.70)	2596(80.30)	5711(68.96)	2968(59.50)	2343(79.97)	5311(67.08)
Urgent, *n* (%)	997(19.75)	430(13.30)	1427(17.23)	45(0.90)	396(13.52)	441(5.57)
Critical, *n* (%)	937(18.56)	207(6.40)	1144(13.81)	975(19.55)	191(6.52)	1166(14.73)
Charlson Comorbidity Index						
0, *n* (%)	2196(43.49)	2143(66.29)	4339(52.39)	2143(42.96)	1032(35.22)	3175(40.10)
1–2, *n* (%)	1208(23.93)	1332(41.20)	2540(30.67)	1332(26.70)	678(23.14)	2010(25.39)
3–4, *n* (%)	1089(21.57)	965(29.85)	2054(24.80)	965(19.35)	585(19.97)	1550(19.58)
≥5, *n* (%)	556(11.01)	548(16.95)	1104(13.33)	548(10.99)	635(21.67)	1183(14.94)
Type of Insurance Coverage						
UEBMI, *n* (%)	1928(38.19)	1631(50.45)	3559(42.97)	1658(33.24)	1440(49.15)	3098(39.13)
URBMI, *n* (%)	325(6.44)	360(11.14)	685(8.27)	259(5.19)	335(11.43)	594(7.50)
NCMS, *n* (%)	1818(36.01)	871(26.94)	2689(32.47)	1928(38.65)	852(29.08)	2780(35.11)
Self-payment, *n* (%)	927(18.36)	263(8.13)	1190(14.37)	1076(21.57)	228(7.78)	1304(16.47)
Other, *n* (%)s	51(1.01)	108(3.34)	159(1.92)	67(1.34)	75(2.56)	142(1.79)
Length of Stay (days), Mean ± SD						
LOS	11.77(6.83)	12.42(7.55)	12.17(7.29)	11.20(6.51)	12.20(7.53)	12.30(7.54)
Total, *n*(%)	5049(60.96)	3233(39.04)	8282	4988(63.00)	2930(37.00)	7918

Note: UEBMI, the urban employee-based basic medical insurance; URBMI, urban resident-based basic medical insurance scheme; NCMS, the rural new cooperative medical scheme; LOS, length of stay; AMI, acute myocardial infarction.

**Table 2:** Descriptive statistics of unadjusted hospitalization expenses for per inpatient over two time periods and by treatment group status.

Variable		Mean	SD	Q10	Q25	Q50	Q75	Q90
Total hospitalization expenses (USD)		3988.25	3829.27	835.77	1357.33	2648.16	6110.53	9253.79
Control group	Pre- intervention period	3914.84	4173.89	811.48	1316.8	2631.87	5531.49	7225.64
	Post- intervention period	3897.23	4901.43	811.13	1324.79	2641.57	5590.81	6885.59
Treated group	Pre- intervention period	4016.77	3454.34	816.24	1350.65	2603.27	5605.8	10414.64
	Post- intervention period	4124.14	3787.42	862.77	1400.43	2732.07	5625.46	10492.77
Drug expenses (USD)		1214.79	1452.39	256.46	478.12	836.09	1472.47	2602.21
Control group	Pre- intervention period	1180.47	1527.3	289.44	494.91	872.72	1505.8	1797.13
	Post- intervention period	1091.45	1335.68	265.17	456.84	787.81	1355.92	1569.33
Treated group	Pre- intervention period	1305.78	1530.12	295.06	523.76	870.76	1507.04	3327.88
	Post- intervention period	1151.49	1416.49	243.6	437.7	735.16	1243.98	2897.46
Diagnosis expenses (USD)		715.76	583.69	233.35	384.06	633.41	877.21	1358.78
Control group	Pre- intervention period	724.79	580.71	246.56	381.16	579.71	852.57	949.67
	Post- intervention period	700.05	538.79	145.12	367.58	586.23	856.67	926.54
Treated group	Pre- intervention period	714.45	575.97	268.41	406.49	608.26	894.38	1529.17
	Post- intervention period	723.73	639.32	271.1	413.19	607.97	872.9	1559.83
Treatment expenses (USD)		660.92	834.24	42.61	124.78	385.65	993.26	1546.35
Control group	Pre- intervention period	618.9	792.47	38.71	105.36	338.48	790.58	1635.78
	Post- intervention period	682.97	850.86	45.23	109.2	365.14	1018.19	1728.51
Treated group	Pre- intervention period	614.61	754.66	39.26	111.92	349.35	945.4	1340.65
	Post- intervention period	727.18	901.47	64.91	165.58	432.83	1060	1549.22
Material expenses (USD)		1308.81	2167.42	26.61	54.72	175.68	2416.68	4336.38
Control group	Pre- intervention period	1310.73	2120.50	24.54	51.46	71.25	1406.42	4057.98
	Post- intervention period	1353.66	2121.41	27.31	55.39	79.06	1681.37	4047.41
Treated group	Pre- intervention period	1336.1	2239.05	23.85	62.68	254.24	1939.97	4684.52
	Post- intervention period	1234.73	2123.52	26.6	65.45	244.33	1582.41	4318.92
Service expenses (USD)		70.47	82.54	9.25	34.31	44.56	119.22	153.51
Control group	Pre- intervention period	79.95	91.33	8.25	37.12	51.35	125.60	161.33
	Post- intervention period	69.1	78.92	5.35	35.72	49.88	89.53	137.25
Treated group	Pre- intervention period	45.83	52.21	3.54	29.91	46.57	67.98	109.56
	Post- intervention period	87.01	95.37	7.89	25.34	71.22	120.50	173.25

**Table 3:** The ZMD policy effects on hospitalization expenses for inpatients from QDID and DID models.

Outcome ^a^	QDID					DID
	Q10	Q25	Q50	Q75	Q90	
	D-I-D	D-I-D	D-I-D	D-I-D	D-I-D	
Total hospitalization expenses	988.32 ***	1805.68 ***	1141.81 ***	602.82 ***	225.83 **	2308.83 ***
SE	(187.31)	(251.12)	(324.01)	(427.60)	(755.59)	(476.41)
Drug expenses	−20.39	−172.93 *	−507.84 ***	−844.77 ***	−1400.00 ***	−661.84 ***
SE	(77.73)	(74.18)	(90.91)	(149.70)	(290.97)	(202.80)
Diagnostics expenses	338.30 ***	247.09 ***	−112.62 *	−96.34	−34.36	206.74 ***
SE	(60.26)	(54.21)	(50.46)	(66.23)	(118.39)	(78.78)
Treatment expenses	334.10 ***	453.01 ***	465.29 ***	267.446 **	609.846 ***	515.74 ***
SE	(34.42)	(47.95)	(52.90)	(85.57)	(162.53)	(99.24)
Material expenses	53.82 ***	43.52 *	54.831	27.69	82.17	54.14
SE	(13.2)	(19.13)	(42.99)	(53.85)	(114.39)	(293.76)
Service expenses	119.16 ***	153.36 ***	61.41 ***	78.04 ***	74.61 **	75.06 **
SE	(36.55)	(30.14)	(9.27)	(17.86)	(31.07)	(24.98)

Note: DID, difference-in-differences estimate; QDID, quantile difference-in-differences estimate; * *p* ≤ 0.05; ** *p* ≤ 0.01; *** *p* ≤ 0.001. Q10, 10th percentile; Q25, 25th percentile; Q50, 50th percentile; Q75, 75th percentile; Q90, 90th percentile. SE, Standard errors. ^a^ Age, gender, disease type, type of insurance coverage, admission status, admission source, Charlson comorbidity index were controlled.

## Data Availability

The datasets analyzed for the current study are available from the corresponding authors on reasonable request.
